# Do Market Characteristics Matter? Factors Associated with Health Information Exchange

**DOI:** 10.3390/ijerph182211976

**Published:** 2021-11-15

**Authors:** Na-Eun Cho, KiHoon Hong, Jongwha Chang

**Affiliations:** 1 Department of Business Administration, School of Business, Hongik University, Seoul 04066, Korea; khhong@hongik.ac.kr; 2 Department of Healthcare Administration, College of Business, Texas Woman’s University, Denton, TX 76204, USA; jchang3@twu.edu

**Keywords:** health information technology, information exchange, hospital, market

## Abstract

This study explores factors associated with the breadth (extent) and depth (level of detail) of digital information exchange among stakeholders in health information technology (IT) systems. Annual and IT surveys of the American Hospital Association and the U.S. Census Bureau’s small-area income and poverty estimates from 2014–2016 were analyzed for associations between key factors and breadth and depth of information exchange. OLS Regression was used with a sample consisting of 10,040 year-hospital observations. We found that hospital-level variables such as size, ownership type, system affiliation, physician-hospital arrangement, and revenue model affect information exchange. We further found that market-level variables such as concentration ratio, urbanness, and median household income, although they directly affect information exchange, do not moderate the relationship between hospital-level variables and information exchange. Our study fills a gap in the previous literature arising from the lack of research on the determinants of health information exchange.

## 1. Introduction

Many economically developed countries have put great effort into implementing health information technology (IT) to reduce healthcare costs and improve quality of care [1,2]. Examples include the Health Information Technology for Economic and Clinical Health (HITECH) Act passed in the U.S., which provided USD 27 billion in incentive payments to encourage adoption of electronic health records over a 10-year period [1], and the U.K. government’s 2018 announcement of an approximately USD 540 million investment in hospital information technology [2]. Although the U.S. Office of the National Coordinator for Health Information Technology (2018) reported 96% of all non-federal acute care hospitals to possess a certified health IT system as of 2017 [3]; however, there exists a gap between health IT adoption and use, specifically with regard to information exchange among healthcare providers. Despite a high rate of health IT adoption, 32% of patients who visited a healthcare provider within the past 12 months reported having to repeat a procedure, provide their medical history again, or bring a test result to an appointment because of the unavailability of prior data [4]. The implication is that there exists many hospitals that have adopted health IT and do not successfully share electronic information among providers.

Scholars, taking note of the importance of information exchange, have begun to focus on how information is incorporated and shared in healthcare settings [5]. A study comparing length of stay and readmission rates between instances of assimilation of electronic health records, measured as achievement of meaningful use and mere adoption, found patient outcomes to improve only in the former case [6]. Another study found that electronic mobilization of healthcare information across organizations could save as much as USD 1.9 million in emergency department settings annually [7]. These results commonly support the idea that it is the actual use of health IT, not mere adoption, that determines the extent of benefits, which a hospital gains from IT investment [8].

Increasing interest notwithstanding, little is known about what affects information exchange in the healthcare setting. Determinants of adoption have been widely studied [9,10,11,12,13], but information exchange has not. Exceptions, such as studies examining determinants of meaningful use [14,15], have been based on early stage of meaningful use that has not involved robust electronic health information exchange. That only the final stage of meaningful use (termed “promoting interoperability” in fall 2018) includes electronic information exchange as a major requirement, and its reporting years are ongoing (2019–2021) [16], suggests that prior studies provide limited understanding of what affects the exchange of health information. Recently, scholars have started examining the factors associated with health information exchange, but they have either focused on a few individual relevant factors such as system membership or incentives [17,18,19] or on one aspect of information shared electronically, e.g., volume [20].

With the goal of providing a more comprehensive picture of drivers of health information exchange, the present study examines specific factors that affect information exchange among health IT systems. We investigate, based on findings reported in the prior literature about their effect on “mere adoption” of health IT [9,10,11,12], whether factors such as size, concentration, system affiliation, ownership type, teaching status, urbanness, etc. affect information exchange similarly or differently. Responding to the increasing importance of information exchange among healthcare providers, as detailed above, our study focuses on configuration strategies found to affect hospital performance [21], specifically, what affects the breadth (i.e., the extent to which patient health information is shared electronically among stakeholders) and depth (i.e., the level of detail at which patient health information is shared electronically) of information exchange among stakeholders.

Using data sets from the American Hospital Association’s (AHA’s) annual and IT surveys, and the Census Bureau’s small-area income and poverty estimates for the 2014–2016 period, we estimate the association of key factors with breadth and depth of information exchange and derive important policy implications from evaluations of previously studied determinants of health IT adoption. The paper is organized as follows. In Section 2, we describe our data and ex-ante predictions. Results are discussed in Section 3. We present our conclusions in Section 4.

## 2. Materials and Methods

We compiled 2014–2016 data from multiple sources including the Annual and IT surveys of the American Hospital Association (AHA) (https://www.ahadata.com/ accessed on 1 March 2021) and U.S. Census Bureau’s small-area income and poverty estimates (https://www.census.gov/ accessed on 1 March 2021). The current research did not need to be reviewed and approved by the institutional review board because these data sets do not involve “human subjects”. Annual AHA surveys include not only hospital-level information, such as bed size, hospital ownership type, teaching status, system affiliation, physician-hospital integration, and revenue models, but also market-level information, such as urbanness and market concentration, and AHA IT surveys provide detailed information regarding breadth and depth of information exchange. The U.S. Census Bureau’s small-area income and poverty estimates provide information about median household income proximate to each hospital’s location.

To examine the association between various factors and breadth/depth of information exchange, we selected 10 determinants of health IT adoption, such as electronic medical records or electronic health records. We chose seven hospital-level (bed size, hospital ownership type (two dummy variables to categorize for-profit, government, and nonprofit), teaching status, system affiliation, physician-hospital integration, and revenue models) and three market-level (concentration, urbanness, and median household income) variables.

### 2.1. Hospital-Level Determinants of Information Exchange

Our ex-ante expectation of the relation of bed size to breadth and depth of information exchange is positive and statistically significant. Just as their more abundant resources make it easier for larger than for smaller hospitals to adopt health IT (i.e., electronic medical records and electronic health records) [10,11,12,22], so superior technical capabilities make it easier for larger hospitals to exchange information electronically with other stakeholders.

Results for ownership type were inconsistent. Previous research has suggested that health IT is more likely to be adopted and implemented by for-profit hospitals with greater financial resources than by nonprofit or government hospitals [11,12,23]. Effects of ownership type, however, were statistically insignificant in one study [12] and significant in another [11], and other studies have found nonprofits, because they view it as part of their public service responsibility, to in fact be more likely than other hospital types to implement health IT [9]. Due to these inconsistencies, effect of ownership type on information exchange is not predicted in our study.

Prior literature also suggests that young medical students, residents, and fellows at teaching hospitals, being more comfortable with new technology, may find it easier to share information electronically with others [11]. Thus, our ex-ante expectation for the effect of a teaching hospital on information exchange is positive and statistically significant. System affiliation is also expected to increase information exchange, affiliated institutions that share organizational practices, culture, and policies [11,18] being more likely to share health information as well. Physician-hospital integration, that is, employment of physicians by hospitals, is expected to provide greater control over physician behavior and thus facilitate hospitals’ implementation of new technology [24]. Thus, our ex-ante expectation for the effect of this variable on information exchange is positive and statistically significant. Different incentive models such as bundled payment or alternative payment models are expected to be one of the key organizational determinants of information exchange [17,19,20]. Similarly, we expect hospitals with capitation model-based revenue, whereby providers receive a fixed per person payment regardless of actual services provided, to be more incentivized to share detailed information in order to reduce overall costs. Our ex-ante expectation of capitation revenue is thus positive and statistically significant.

### 2.2. Market-Level Determinants of Information Exchange

We measured concentration, one of the environmental variables included in our study, as the Herfindahl index. Hospitals facing greater competition with attendant pressure for cost-reduction, although they might be expected to put greater effort into actively sharing information electronically, may find it difficult to do so owing to differences in IT system vendors or technical capabilities across providers and hospitals. This might explain the statistical insignificance of the effect of competition in a previous study [11]. Our ex-ante expectation of the effect of competition on information exchange is thus not predicted.

The ex-ante expectation of the effect of urbanness on information exchange is negative and statistically significant. Whereas previous literature has suggested that urban hospitals with greater resources are likely to adopt health IT [20], once equipped with IT systems, rural hospitals that share similar cultures are likely to find it easier to share patient information. That the ex-ante prediction of the relation between munificence and information exchange is positive and statistically significant corroborates our expectation that hospitals located in affluent areas, other things being constant, are more likely to appeal to patients by more effectively employing information technology, such as electronic medical records or electronic health records.

### 2.3. Measurement

We employed a multiple regression analysis with 10 variables, as follows.

(1) $$\begin{array}{c} Information\;Exchange_{it}\\ \;\;\;\;\;\;\;=\alpha +\beta_{1}Bed\;Size_{it}\\ \;\;\;\;\;\;\;+\beta_{2}For-profit\;Hospital_{it}+\beta_{3}Government\;Hospital_{\;it}+\beta_{4}Teaching\;Hospital_{it}\\ \;\;\;\;\;\;\;+\beta_{5}System\;Affiliation_{it}+\beta_{6}Physician-Hospital\;Integration_{it}\\ \;\;\;\;\;\;\;+\beta_{7}Capitation\;Revenue_{it}+\beta_{8}HHI_{it}+\beta_{9}Urvanness_{it}+\beta_{10}Median\;Household\;Income_{it}\\ \;\;\;\;\;\;\;+Year_{t}+\in_{it} \end{array}$$

Our main dependent variable, information exchange, was measured in two ways, as breadth and depth of information exchange [21]. The breadth variable was measured based on answers to questions about whether a hospital provides or electronically shares data with (1) other hospitals in its system, (2) hospitals outside its system, (3) ambulatory providers in its system, and (4) ambulatory providers outside its system. The depth variable was measured based on answers to questions about how much detailed data is shared (five categories: patient demographics, laboratory results, medication history, radiology reports, clinical/summary care records). The values of each item were summed to generate the breadth and depth variables. While we noticed that breadth and depth of information exchange have been identified differently in previous literature [17,19], we decided to use the definition and measurement of breadth and depth following the paper that first operationalized these concepts using AHA IT surveys, to the best of our knowledge [21].

Bed size is the total number of hospital beds, and for-profit hospital and government hospital are dummy variables. Both dummies equal to zero signify a voluntary nonprofit hospital. Teaching hospital is a dummy variable that takes the value of 1 if the hospital is a teaching hospital. System affiliation is also a dummy variable. Physician-hospital integration is a binary variable that takes the value of 1 if a hospital has an arrangement whereby physicians become employees of the hospital, that is, employs an integrated salary model, and 0 otherwise [24,25]. Previous literature has shown the integration salary model to be the tightest mode of integration between physicians and hospitals. Capitation revenue is also a dummy variable that takes the value of 1 if a hospital’s net revenue paid on a capitated basis or shared risk basis. We expect physicians and hospitals under predetermined basis to be encouraged to reduce overall costs and avoid unnecessary tests or procedures. HHI is the Herfindahl–Hirschman index, based on total facility admissions. A high HHI index implies that a hospital is located in a concentrated market, a low HHI index that a hospital is in a competitive market. Urbanness is a dummy variable that takes the value of 1 if a hospital is located in an urban area, and 0 if located in a rural area. Median household income is a county-level variable that describes an area’s munificence.

## 3. Empirical Results

Table 1 reports descriptive statistics for the variables for 10,040 year-hospital observations (3258 in 2014, 3495 in 2015, and 3597 in 2016). According to Table 1, 19% of our observations are for-profit, 22% government, and the remainder nonprofit hospitals.

Table 2 shows the results of our ordinary least squares (OLS) regression analyses regarding the effect of the 10 aforementioned variables on breadth and depth of information exchange. To ensure that our models do not suffer from multicollinearity, we conducted the variance inflation factor (VIF) test. Our model has a VIF of 1.38, indicating that there is not enough evidence to determine that our model suffers from serious multicollinearity problem.

The estimated coefficients of the variables’ bed size, system affiliation, physician–hospital integration, and capitation revenue model, being consistent with our stated ex-ante expectations, are not discussed further. Results on the effect of ownership type, not predicted in our study, indicate nonprofit to be more likely than either for-profit or government owned hospitals to share detailed information with other stakeholders. This suggests that nonprofits, being prohibited by law from making a commercial or monetary profit, take information exchange more seriously than other hospital types owing to altruistic concerns about quality. The coefficient of teaching hospital is insignificant, suggesting that the inclination to exchange information electronically is not greater among those working at teaching hospitals than among those employed by non-teaching hospitals. This might reflect lack of other organizations’ ability to engage in electronic exchange despite teaching hospitals’ interest in sharing information with outside stakeholders. The effect of HHI, which is not predicted in our study, is positive and statistically significant, suggesting that hospitals in a given county with limited competition are more likely than those in highly competitive areas to share detailed information with others. The estimated coefficient of the effect of urbanness on depth of information exchange is consistent with our stated ex-ante expectation, while the one on breadth of information exchange is not. One possible explanation for the difference is that shortage of financial resources may offset the information sharing effect originated by similar cultures in rural areas. The coefficient of median household income is small but positive, consistent with our ex-ante prediction.

Examining hospital and market-level determinants of health IT-driven information exchange addresses a gap in previous literature that has not looked carefully at the actual use of health IT. More interesting, perhaps, is that although hospital-level determinants are more amenable from the perspective of hospital administrators and policymakers, market-level determinants are more or less deterministic. Whereas hospitals can, for example, increase or reduce numbers of beds or alter arrangements with physicians, they are unlikely to move from an urban to a rural area or be able to effect a change in the overall income level of the county in which they are situated.

This leads us to further investigate whether effects of hospital-level determinants vary with market characteristics. To this end, we divide the full sample into two groups according on HHI, Urban/Rural, and median household income, and for HHI and median household income split the sample by taking above and below the mean. Results are reported in Table 3 and Table 4. The results of VIF test suggest that there is not enough empirical evidence to determine that our models suffer from serious multicollinearity (Mean VIF: 1.27 (column 1), 1.23 (column 2), 1.27 (column 3), 1.14 (column 4), 1.27 (column 5), and 1.28 (column 6) in Table 3 and Table 4, respectively).

Estimated coefficients of most hospital-level variables, including bed size, ownership type, teaching status, system affiliation, and physician-hospital integration, are the same for the sub-sample as for the full sample analysis. These results suggest that market-level variables, although they seem to affect information exchange directly, do not indirectly affect hospital-level variables’ influence on information exchange. The results further imply that government does not necessarily need to modify policy according to market characteristics to encourage information exchange among providers.

The coefficient of capitation revenue differs by market characteristics, for breadth of information exchange becoming insignificant in highly concentrated and rural areas, and for depth of information exchange becoming insignificant in highly concentrated, rural, and low-income areas. A possible explanation for this result is that hospitals located in competitive, urban, high-income regions face more pressure to provide high-quality services based on health IT. As this aspect was beyond the scope of our study, we leave exploration of this possibility to future research.

## 4. Discussion

Our study makes a number of contributions. First, to the best of our knowledge, the current study is the first to investigate determinants of information exchange in the healthcare setting. Previous research has focused mostly on determinants of adoption, and in such studies of meaningful use as existing information exchange has only recently been added as a requirement [17,18,19,20]. Understanding of the determinants of information exchange has thus been limited. The results of our study should contribute to the realization of many of the benefits of health IT, including those associated with enhanced information exchange.

Second, our study helps to provide a more comprehensive picture of drivers of health information exchange. Rather than merely focusing on the percentage of information shared electronically, the current study focused on the relevant factors associated with the breadth and depth of information shared. Moreover, our study examines one of the key hospital-level factors not previously studied: physician-hospital integration. Even among hospital-level variables, it is relatively easier to change physician-hospital arrangements than to change, for example, ownership type. Those interested in enhancing information exchange should continue to search for other unexamined factors that might increase cross-party sharing of detailed information.

Last but not least, our analysis elucidates the effect of specific market-level determinants on information exchange. The finding of our sub-sample analysis that market-level variables such as concentration, urbanness, and median household income do not moderate the relation between hospital-level variables and information exchange is particularly important, as hospital-level determinists are more amenable and hospital administrators and policymakers thus do not need to pursue different strategies to promote the widespread use of health IT using the results of our study. The government with limited resources should thus revisit policies of increasing health information exchange and focus more on hospital-level determinants, such as revenue model or system affiliation.

Few limitations of our paper could shed light on noble opportunities for future research. In examining the determinants of information exchange, the current study only looks at two dimensions: the breadth (extent) and depth (level of detail). Recent studies suggests that four dimensions, including volume, diversity, breadth, and depth are important [17,19]. Future studies could examine the factors we studied that could also affect other dimensions of information exchange, and the varying effect by market characteristics. Furthermore, while our study focuses on the three market-level variables, HHI, urbanness, and median household income, the future researchers can examine other social contexts, such as race, patient mix, and percentage of those who receive care in an integrated system (e.g., Health Maintenance Organization).

## 5. Conclusions

We analyzed multiple years of national data to inform understanding of the use of health IT, specifically, information exchange, beyond mere adoption. Aided by detailed datasets rarely used previously, our study examines the relationship between select unexamined variables and breadth and depth of information exchange in healthcare settings. We found that hospital-level variables such as size, ownership type, system affiliation, physician-hospital arrangement, and revenue model affect information exchange, and that market-level variables such as concentration ratio, urbanness, and median household income, although they directly affect information exchange, do not moderate the relationship between hospital-level variables and information exchange.

This paper enhances understanding of factors associated with information exchange via health IT and its implications for the sustainability of the healthcare system. Efforts in many countries to increase information exchange using health IT effectively have gained added importance during the present pandemic. Among the insights related to theory and policy yielded by our results is that hospital administrators and policymakers who seek to increase health IT-driven information exchange should focus on hospital-level determinants and not differentially formulate strategies according to market-level characteristics.

## Figures and Tables

**Table 1:** Descriptive Statistics.

Variable	Mean	SD	Min	Max
Breadth	2.815	1.393	0	4
Depth	4.323	1.516	0	5
Bed Size	174.074	205.660	1	2829
For-profit Hospital	0.191	0.393	0	1
Government Hospital	0.224	0.417	0	1
Teaching	0.936	0.244	0	1
System Affiliation	0.638	0.481	0	1
Physician-Hospital Integration	0.437	0.496	0	1
Capitation	0.054	0.226	0	1
HHI	0.608	0.348	0.025	1
Urbanness	0.656	0.475	0	1
Median Household Income	53,668.670	13,873.250	22,640	134,609

**Table 2:** Hospital- and Market-level Determinants of Information Exchange.

	(1)	(2)
VARIABLES	Breadth	Depth
Bed Size	0.001 ***	0.001 ***
	(0.000)	(0.000)
For-profit Hospital	−0.979 ***	−1.022 ***
	(0.054)	(0.065)
Government Hospital	−0.495 ***	−0.335 ***
	(0.047)	(0.052)
Teaching	0.041	−0.029
	(0.066)	(0.057)
System Affiliation	0.577 ***	0.411 ***
	(0.037)	(0.043)
Physician-hospital Integration	0.223 ***	0.109 ***
	(0.033)	(0.036)
Capitation	0.189 ***	0.153 ***
	(0.061)	(0.049)
HHI	0.254 ***	0.205 ***
	(0.060)	(0.066)
Urbanness	0.015	−0.155***
	(0.048)	(0.052)
Median Household Income	0.000 *	0.000
	(0.000)	(0.000)
Constant	1.994 ***	3.902 ***
	(0.100)	(0.112)
Observations	10,040	10,040
R-squared	0.200	0.119

Standard errors (in parenthesis) are clustered at the hospital level; *** *p* < 0.01, * *p* < 0.1.

**Table 3:** Sub-sample Analysis—Breadth of Information Exchange.

	(1)	(2)	(3)	(4)	(5)	(6)
DV: Breadth of Information Exchange	Low HHI	High HHI	Urban	Rural	Low Median Household Income	High Median Household Income
Bed Size	0.001 ***	0.001 ***	0.001 ***	0.002 ***	0.001 ***	0.001 ***
	(0.000)	(0.000)	(0.000)	(0.000)	(0.000)	(0.000)
For-profit Hospital	−1.011 ***	−0.987 ***	−1.025 ***	−0.925 ***	−1.003 ***	−1.026 ***
	(0.068)	(0.084)	(0.061)	(0.120)	(0.074)	(0.073)
Government Hospital	−0.514 ***	−0.479 ***	−0.498 ***	−0.453 ***	−0.411 ***	−0.541 ***
	(0.078)	(0.056)	(0.069)	(0.063)	(0.059)	(0.069)
Teaching	0.046	0.026	0.032	−0.179	−0.056	0.060
	(0.071)	(0.148)	(0.066)	(0.582)	(0.114)	(0.075)
System Affiliation	0.610 ***	0.548 ***	0.584 ***	0.554 ***	0.615 ***	0.515 ***
	(0.055)	(0.049)	(0.049)	(0.058)	(0.051)	(0.053)
Physician-hospital Integration	0.240 ***	0.213 ***	0.250 ***	0.195 ***	0.248 ***	0.216 ***
	(0.047)	(0.045)	(0.042)	(0.054)	(0.047)	(0.046)
Capitation	0.197 **	0.144	0.180 ***	0.175	0.170 *	0.175 **
	(0.076)	(0.095)	(0.066)	(0.141)	(0.101)	(0.070)
Constant	2.212 ***	2.371 ***	2.291 ***	2.295 ***	2.243 ***	2.385 ***
	(0.063)	(0.058)	(0.057)	(0.068)	(0.058)	(0.061)
Observations	4950	5090	6583	3457	5040	5000
R-squared	0.236	0.158	0.226	0.128	0.191	0.198

Standard errors (in parenthesis) are clustered at the hospital level; *** *p* < 0.01, ** *p* < 0.05, * *p* < 0.1.

**Table 4:** Sub-sample Analysis—Depth of Information Exchange.

	(1)	(2)	(3)	(4)	(5)	(6)
DV: Depth of Information Exchange	Low HHI	High HHI	Urban	Rural	Low Median Household Income	High Median Household Income
Bed Size	0.001 ***	0.001 ***	0.001 ***	0.001 ***	0.001 ***	0.001 ***
	(0.000)	(0.000)	(0.000)	(0.000)	(0.000)	(0.000)
For-profit Hospital	−1.105 ***	−0.996 ***	−1.097 ***	−0.831 ***	−1.095 ***	−1.071 ***
	(0.081)	(0.103)	(0.073)	(0.138)	(0.091)	(0.089)
Government Hospital	−0.377 ***	−0.290 ***	−0.406 ***	−0.235 ***	−0.170 ***	−0.453 ***
	(0.085)	(0.063)	(0.076)	(0.070)	(0.063)	(0.081)
Teaching	-0.046	0.040	−0.042	0.044	−0.139	0.013
	(0.060)	(0.115)	(0.057)	(0.164)	(0.095)	(0.065)
System Affiliation	0.468 ***	0.332 ***	0.457 ***	0.327 ***	0.409 ***	0.367 ***
	(0.066)	(0.055)	(0.059)	(0.061)	(0.057)	(0.062)
Physician-hospital Integration	0.121 **	0.109 **	0.144 ***	0.056	0.098 *	0.146 ***
	(0.050)	(0.049)	(0.045)	(0.059)	(0.051)	(0.049)
Capitation	0.161 ***	0.106	0.138 **	0.151	0.103	0.141 **
	(0.061)	(0.078)	(0.054)	(0.108)	(0.077)	(0.057)
Constant	3.944 ***	4.164 ***	3.963 ***	4.148 ***	4.054 ***	4.084 ***
	(0.073)	(0.068)	(0.067)	(0.079)	(0.065)	(0.071)
Observations	4950	5090	6583	3457	5040	5000
R-squared	0.159	0.074	0.154	0.046	0.104	0.129

Standard errors (in parenthesis) are clustered at the hospital level; *** *p* < 0.01, ** *p* < 0.05, * *p* < 0.1.

## Data Availability

Annual and IT surveys of the American Hospital Association (AHA) were purchased by both N.-E.C. and J.C. at https://www.ahadata.com/ (accessed on 1 March 2021). The U.S. Census Bureau’s small-area income and poverty estimates that provide information about poverty ratio are publicly available (https://www.census.gov/ (accessed on 1 March 2021).

## Abbreviations

A list of abbreviations is provided as follows: IT (information technology); AHA (American Hospital Association); HHI (Herfindahl-Hirschman Index); OLS (ordinary least squares); VIF (variance inflation factor (VIF).

## References

[1] Miliard M. 10 Years on from Meaningful Use, Major Progress Despite the Challenges. https://www.healthcareitnews.com/news/10-years-meaningful-use-major-progress-despite-challenges.

[2] Miliard M. NHS to Receive £487 m Technology Boost. https://www.theguardian.com/society/2018/jul/20/nhs-to-receive-487m-technology-boost-matt-hancock.

[3] Office of the National Coordinator for Health Information Technology ‘Percent of Hospitals, By Type, that Possess Certified Health IT’, Health IT Quick-Stat #52. September 2018. https://www.healthit.gov/data/quickstats/percent-hospitals-type-possess-certified-health-it.

[4] Office of the National Coordinator for Health Information Technology Gaps in individuals’ information exchange, Health IT Quick-Stat #56. https://www.healthit.gov/data/quickstats/gaps-individuals-information-exchange.

[5] Cresswell K., Sheikh A. (2013). Organizational issues in the implementation and adoption of health information technology innova-tions: An interpretative review. Int. J. Med Inform..

[6] Wani D., Malhotra M. (2018). Does the meaningful use of electronic health records improve patient outcomes?. J. Oper. Manag..

[7] Frisse M.E., Johnson K.B., Nian H., Davison C.L., Gadd C.S., Unertl K., Turri P.A., Chen Q. (2012). The financial impact of health information exchange on emergency department care. J. Am. Med. Inform. Assoc..

[8] Devaraj S., Kohli R. (2003). Performance Impacts of Information Technology: Is Actual Usage the Missing Link?. Manag. Sci..

[9] Ford E.W., Menachemi N., Huerta T.R., Yu F. (2010). Hospital IT adoption strategies associated with implementation success: Im-plications for achieving meaningful use. J. Healthc. Manag..

[10] Hanna T.H., McDowell J.M. (1984). The determinants of technology adoption: The case of the banking firm. Rand J. Eco-Nomics.

[11] Wang B.B., Wan T.T.H., Burke D.E., Bazzoli G.J., Lin B.Y.J. (2005). Factors Influencing Health Information System Adoption in American Hospitals. Health Care Manag. Rev..

[12] Kazley A.S., Ozcan Y.A. (2007). Organizational and Environmental Determinants of Hospital EMR Adoption: A National Study. J. Med Syst..

[13] Kruse C.S., Mileski M., Alaytsev V., Carol E., Williams A. (2015). Adoption factors associated with electronic health record among long-term care facilities: A systematic review. BMJ Open.

[14] Cohen G.R., Adler-Milstein J. (2016). Meaningful use care coordination criteria: Perceived barriers and benefits among primary care providers. J. Am. Med Inform. Assoc..

[15] Green L.A., Potworowski G., Day A. (2015). Sustaining ’meaningful use’ of health information technology in low-resource practices. Ann. Fam. Med..

[16] Center for Medicare & Medicaid Services Promoting Interoperability Programs. https://www.cms.gov/Regulations-and-Guidance/Legislation/EHRIncentivePrograms.

[17] Lin S.C., Hollingsworth J.M., Adler-Milstein J. (2019). Alternative payment models and hospital engagement in health information exchange. Am. J. Manag. Care.

[18] Opoku-Agyeman W., Menachemi N. (2016). Are there differences in health information exchange by health system type?. Health Care Manag. Rev..

[19] Young C.G., Feldman S.S., Hernandez S.R. (2021). Inter-organizational information sharing and bundled payment reimburse-ment: Do hospitals in the US use health information exchange to collaborate?. Int. J. Med. Inform..

[20] Lin S.C., Everson J., Adler-Milstein J. (2018). Technology, Incentives, or Both? Factors Related to Level of Hospital Health Information Exchange. Health Serv. Res..

[21] Cho N., Weiling K., Atems B., Chang J. (2018). How does electronic health information exchange affect hospital performance effi-ciency? The effects of breadth and depth of information sharing. J. Healthc. Manag..

[22] Boonstra A., Broekhuis M. (2010). Barriers to the acceptance of electronic medical records by physicians from systematic review to taxonomy and interventions. BMC Health Serv. Res..

[23] Diana M.L., Harle C.A., Huerta T., Ford E., Menachemi N. (2014). Hospital Characteristics Associated With Achievement of Meaningful Use. J. Health Manag..

[24] Lammers E. (2013). The effect of hospital-physician integration on health information technology adoption. Health Econ..

[25] Cho N.-E., Chang J., Atems B. (2014). The effects of health information technology adoption and hospital-physician integration on hospital efficiency. Am. J. Manag. Care.

